# Supplementary material for: Cas9-induced large deletions and small indels are controlled in a convergent fashion
Source: Nat Commun. 2022 Jun 14;13:3422. doi: 10.1038/s41467-022-30480-8 (PMC9197861; doi:10.1038/s41467-022-30480-8)
Supplement: Supplementary file 1 — Supplementary Information [file 41467_2022_30480_MOESM1_ESM.pdf]

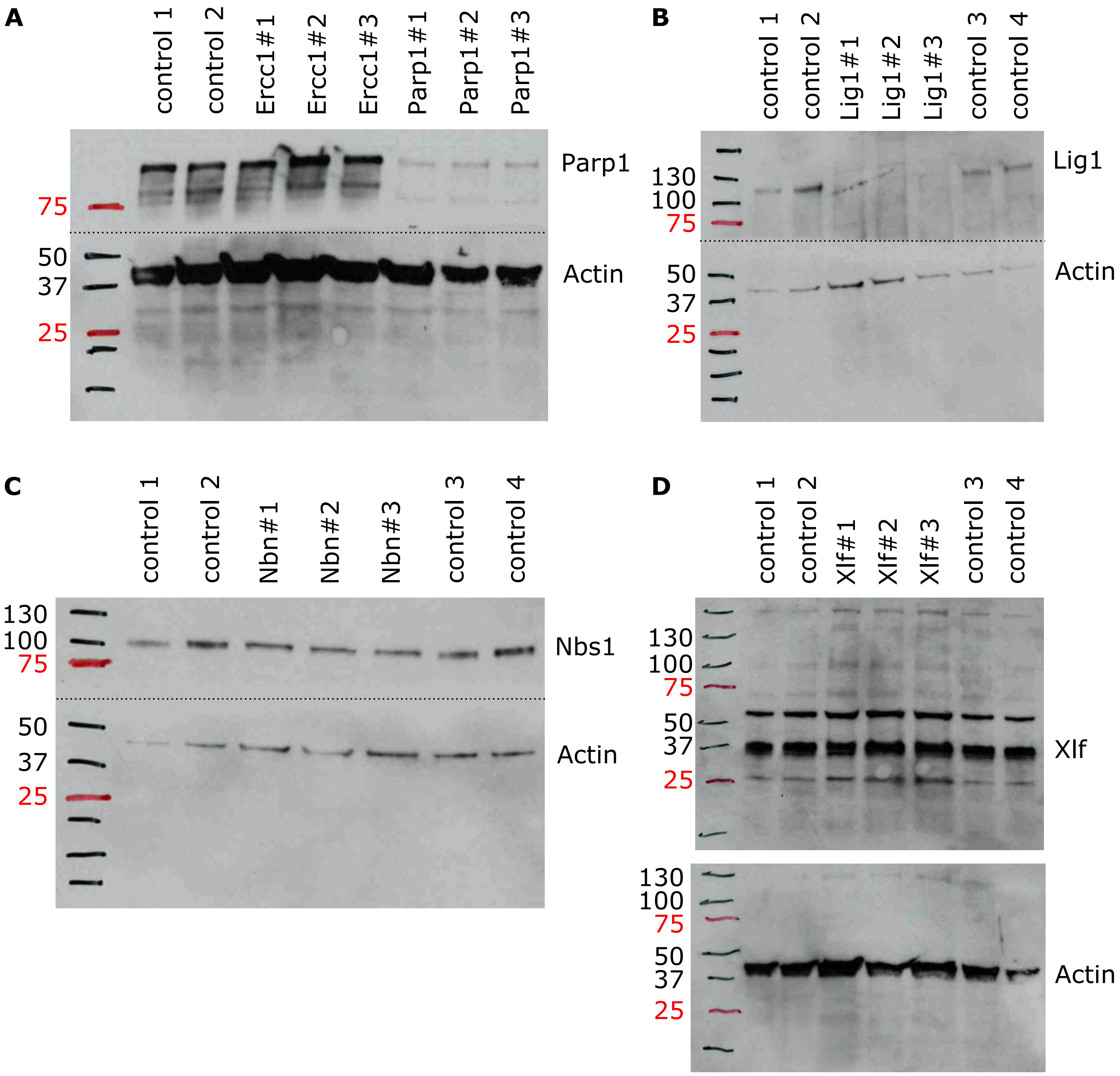

Supplementary Fig. 1. Western blots. (**A**) Parp1 (ab191217), (**B**) Lig1 (18051-1-AP), (**C**), Nbs1 (product of *Nbn* gene; A301–284A) and (**D**) Xlf (A300-730A). Actin (SC-47778) levels were measured either after stripping the original antibody (Xlf) or on the bottom part of the blot (remaining antibodies). Dots indicate the approximate cut line. N=1, molecular marker unit is kDa.

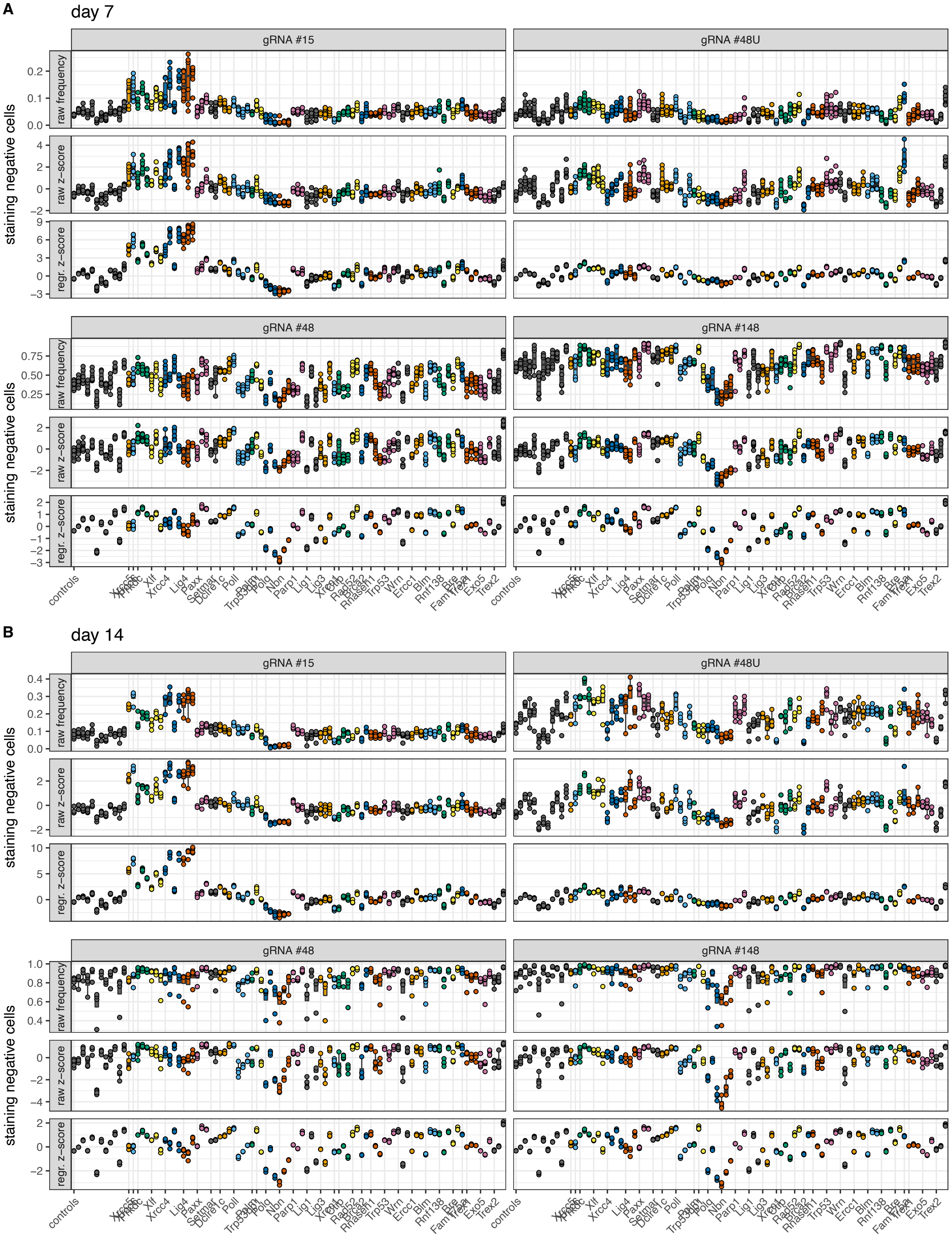

Supplementary Fig. 2. Gene expression negative cells - raw frequency, raw z-score and PCA-regressed z-score. (**A**) Day 7 post-transfection. (**B**) Day 14. Same display conventions as in Fig.1B.

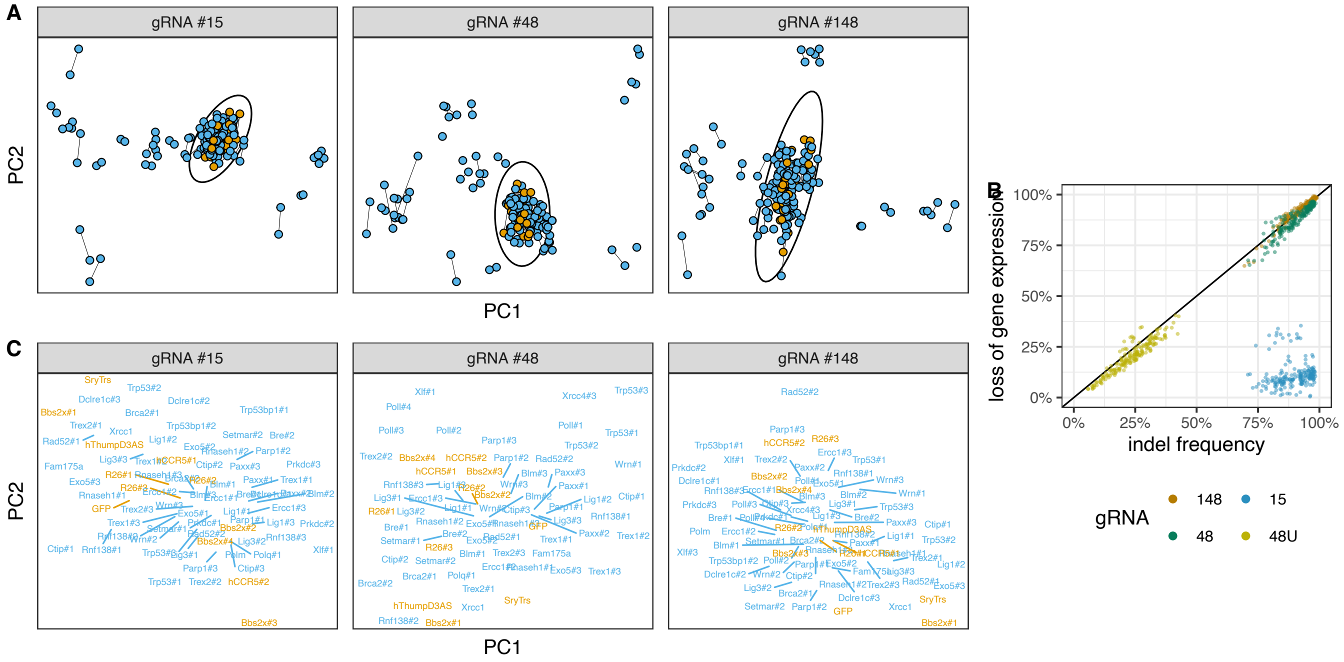

Supplementary Fig. 3. Assay reproducibility and relationships between non-significant clones. (**A**) Relationships between replicate measurements of indel profiles. Ellipse is 99% confidence interval of a bivariate normal distribution fitted to control clones (orange points). (**B**) Comparison of the frequency of mutated alleles as measured by targeted sequencing of short-range PCR products and flow cytometric measurement of protein expression. NB: only large deletions and complex rearrangements, and not small indels, result in loss of gene expression with intronic gRNA #15 (**C**) Relationships between clones not significantly different from controls, compare Fig.2B. Control clones are in orange. Biological replicates (N=2) were averaged.

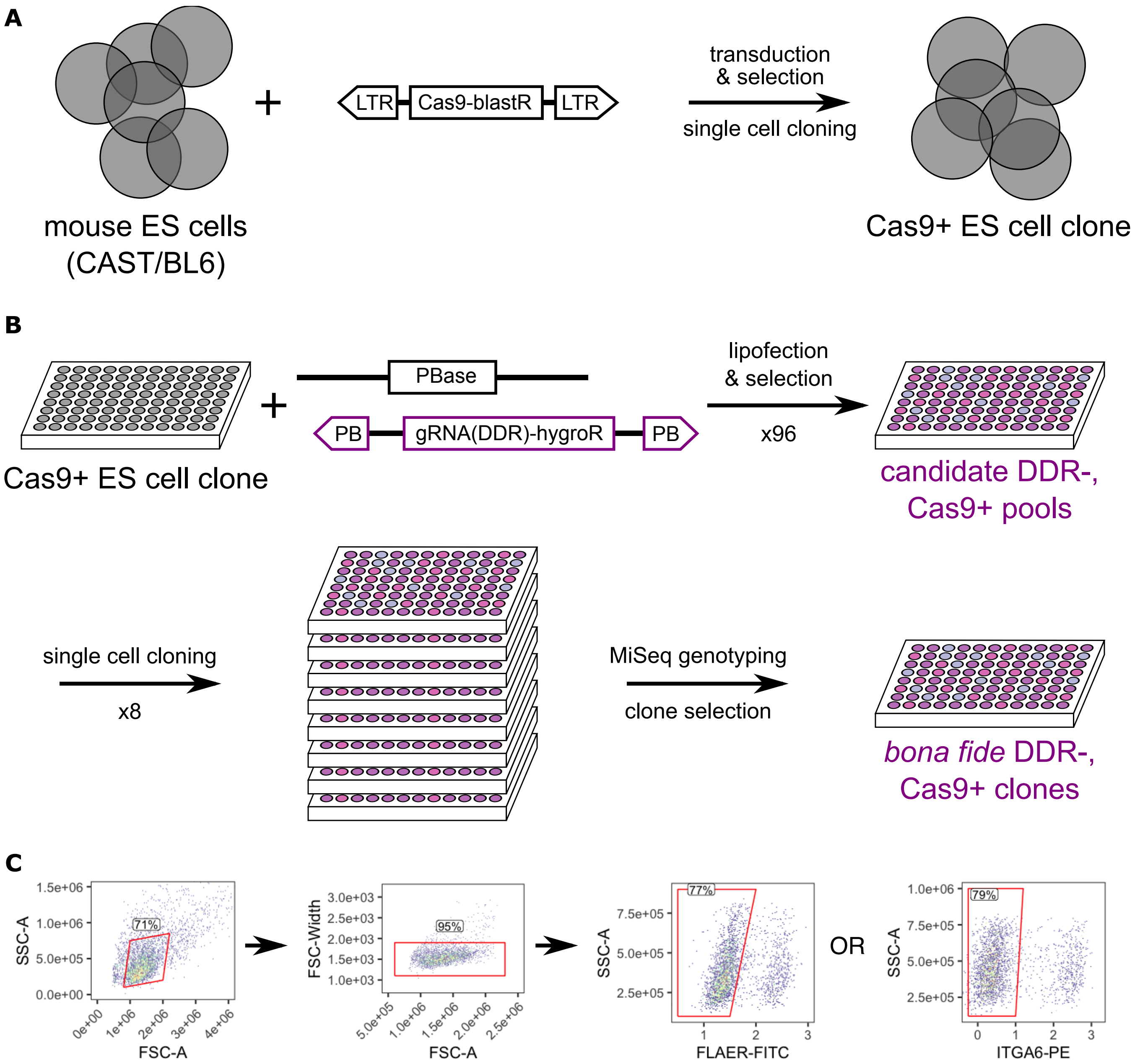

Supplementary Fig. 4. Preparation of Cas9+ ES cell line and library of clones *bona fide* deficient for DNA repair genes and flow cytometry gating. (**A**) Mouse ES cells derived from a cross between CAST and C57BL/6 strains were transduced with a lentivirus containing Cas9 transgene and blastidicin resistance gene, selected and single cell cloned. A clone with highest efficiency of Cas9 editing and no karyotypic abnormalities was selected as the basis for the creation of DNA repair deficient library. (**B**) Cas9+ ES cell clone was transfected in parallel with *PiggyBac* constructs containing gRNAs targeting exons of DNA repair genes (or negative control gRNAs) and a hygromycin resistance gene. Stable transfectants were selected for and single cell cloned, with an average eight clones retained per gRNA. Targeted sequencing of gRNA sites was used to select clones *bona fide* deficient for DNA repair genes based on lack of small in-frame and wild-type indels. (**C**) Gating strategy for the flow cytometric assay.

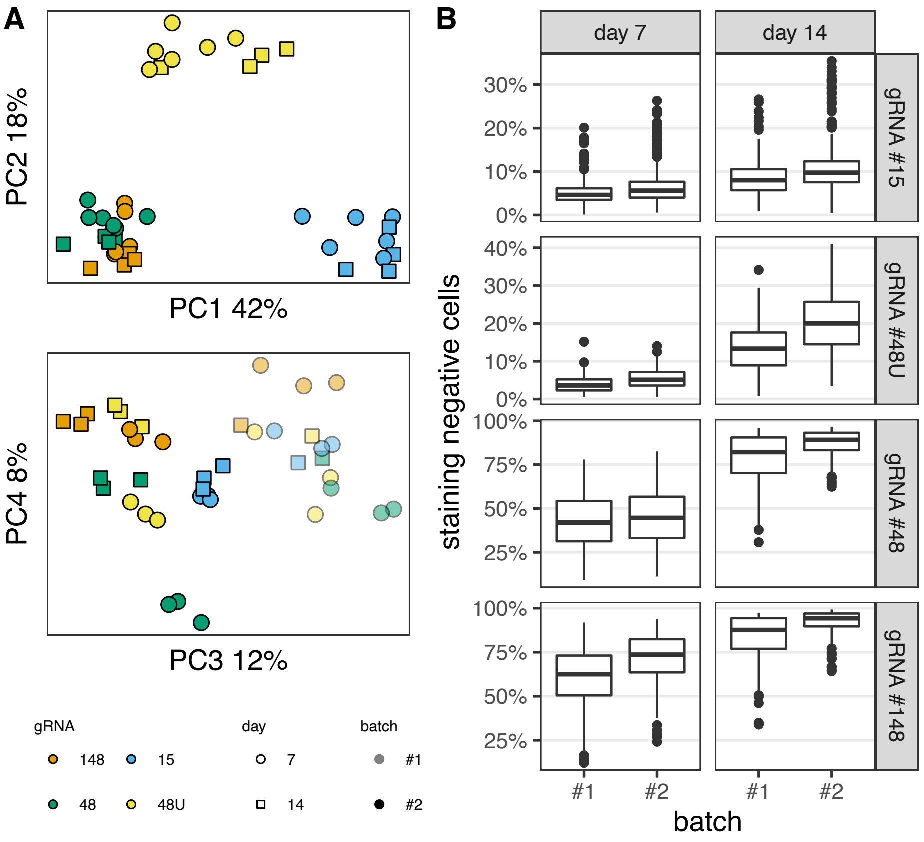

Supplementary Fig. 5. Batch effect in the gene expression assay. (**A**) Relationships between samples based on surface gene expression. (**B**) Frequency of cells negative for the expression of target genes, broken down by batch. Boxplot hinges span IQR (0.25-0.75), line in the middle is median, whiskers extend to the largest value no further than 1.5 * IQR from the hinge. Points beyond that are plotted individually. N=95 for all batch #1 day 14, N=283 for batch #1 day 7 gRNA #48U, N=284 for batch #1 day 7 and batch #2 day 14 gRNA #15 and N=285 for remaining samples. Each clone/replicate is an independent point.

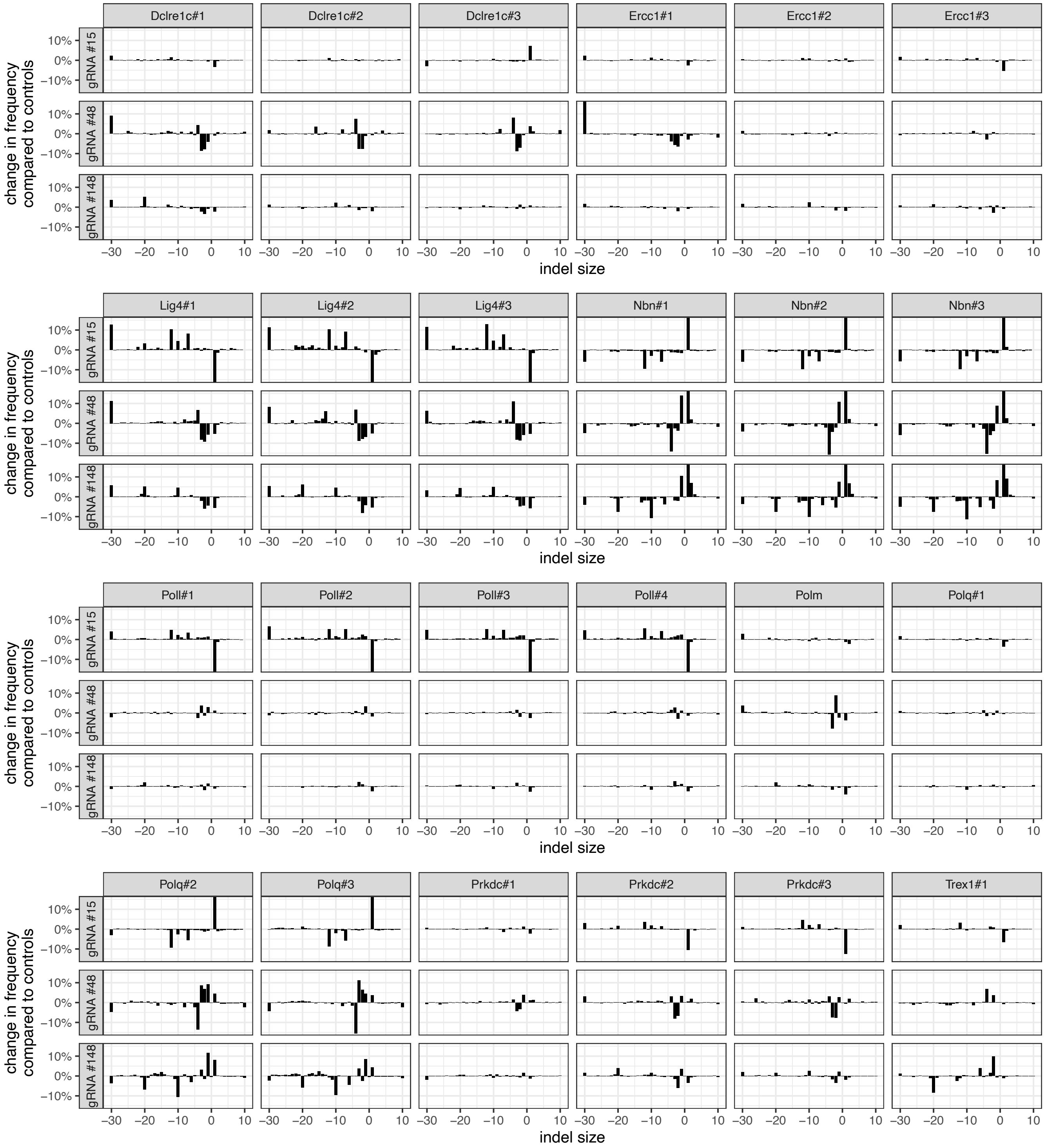
Dclre1c#1
Dclre1c#2
Dclre1c#3
Ercc1#1
Ercc1#2
Ercc1#3
Lig4#1
Lig4#2
Lig4#3
Nbn#1
Nbn#2
Nbn#3
Poll#1
Poll#2
Poll#3
Poll#4
Polm
Polq#1
Polq#2
Polq#3
Prkdc#1
Prkdc#2
Prkdc#3
Trex1#1
gRNA #15
gRNA #48
gRNA #148
10%
0%
−10%
change in frequency
compared to controls
−30
−20
−10
0
10
indel size

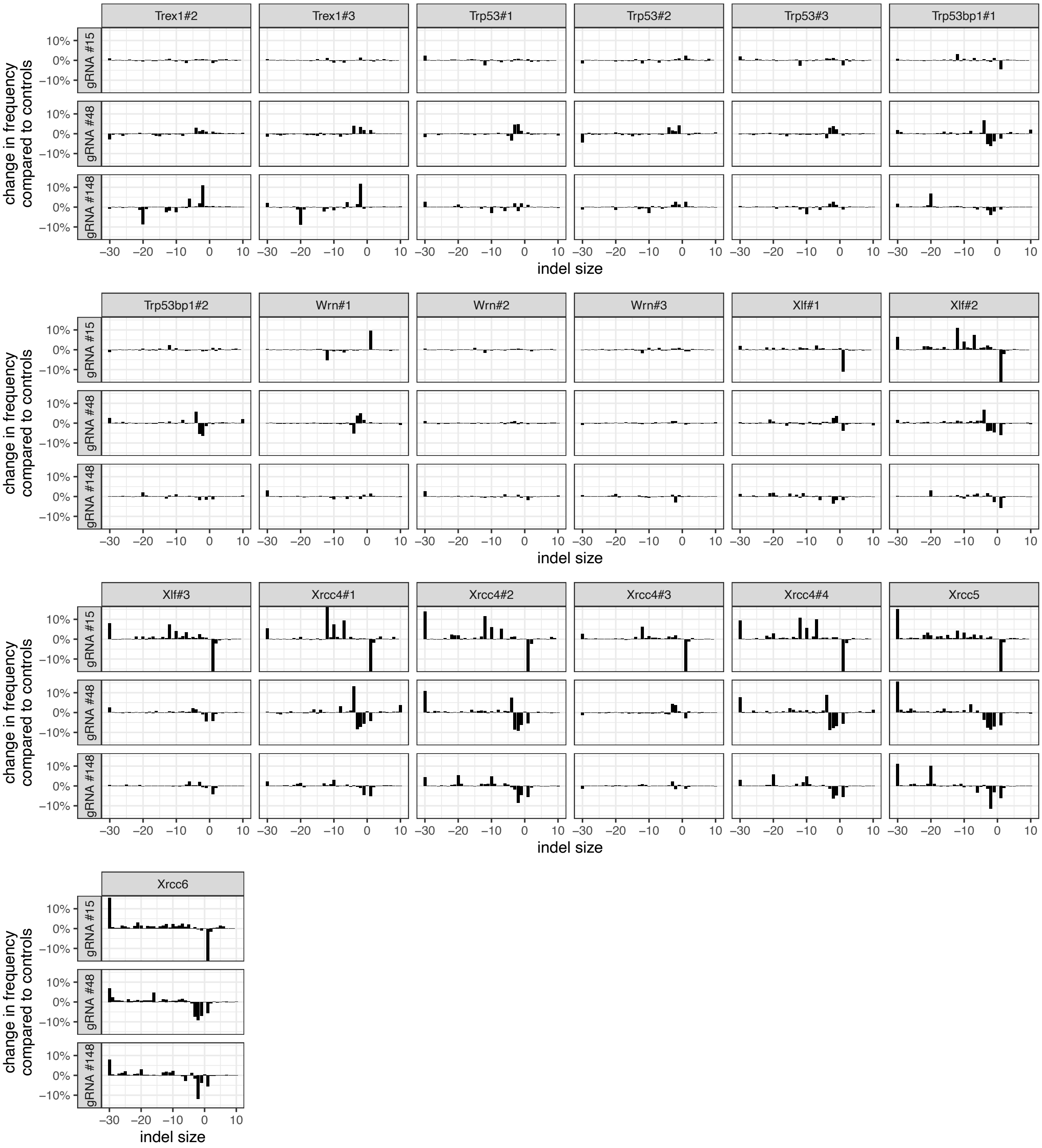

Supplementary Fig. 6. Indel profiles of genes with at least one significant clone. Y-axis is truncated at -15% and +15% for clarity. Figure spans two pages. Same display conventions as in Fig.2.
