# Supplementary material for: Cas9-induced large deletions and small indels are controlled in a convergent fashion
Source: Nat Commun. 2022 Jun 14;13:3422. doi: 10.1038/s41467-022-30480-8 (PMC9197861; doi:10.1038/s41467-022-30480-8)
Supplement: Supplementary file 2 — Reporting Summary [file 41467_2022_30480_MOESM2_ESM.pdf]

nature portfolio

Corresponding author(s): Allan Bradley

Last updated by author(s): Apr 12, 2022

Reporting Summary

Nature Portfolio wishes to improve the reproducibility of the work that we publish. This form provides structure for consistency and transparency in reporting. For further information on Nature Portfolio policies, see our Editorial Policies and the Editorial Policy Checklist.

Statistics

For all statistical analyses, confirm that the following items are present in the figure legend, table legend, main text, or Methods section.

n/a	Confirmed	
[ ]	[x]	The exact sample size (*n*) for each experimental group/condition, given as a discrete number and unit of measurement
[ ]	[x]	A statement on whether measurements were taken from distinct samples or whether the same sample was measured repeatedly
[ ]	[x]	The statistical test(s) used AND whether they are one- or two-sided<br>*Only common tests should be described solely by name; describe more complex techniques in the Methods section.*
[ ]	[x]	A description of all covariates tested
[ ]	[x]	A description of any assumptions or corrections, such as tests of normality and adjustment for multiple comparisons
[ ]	[x]	A full description of the statistical parameters including central tendency (e.g. means) or other basic estimates (e.g. regression coefficient) AND variation (e.g. standard deviation) or associated estimates of uncertainty (e.g. confidence intervals)
[ ]	[x]	For null hypothesis testing, the test statistic (e.g. *F*, *t*, *r*) with confidence intervals, effect sizes, degrees of freedom and *P* value noted<br>*Give P values as exact values whenever suitable.*
[x]	[ ]	For Bayesian analysis, information on the choice of priors and Markov chain Monte Carlo settings
[x]	[ ]	For hierarchical and complex designs, identification of the appropriate level for tests and full reporting of outcomes
[ ]	[x]	Estimates of effect sizes (e.g. Cohen's *d*, Pearson's *r*), indicating how they were calculated

Our web collection on statistics for biologists contains articles on many of the points above.

Software and code

Policy information about availability of computer code

Data collection	Cytoflex software ca. 2017, MiSeq software ca 2018
Data analysis	R 3.6.3-4.1.1 with magrittr_2.0.2, patchwork_1.1.1, scales_1.1.1, ggrepel_0.9.1, cowplot_1.1.1, forcats_0.5.1, stringr_1.4.0, dplyr_1.0.8, purrr_0.3.4, readr_2.1.2, tidyr_1.2.0, tibble_3.1.6, ggplot2_3.3.5, tidyverse_1.3.1. Code: https://gitlab.com/lotard/medraka_paper

For manuscripts utilizing custom algorithms or software that are central to the research but not yet described in published literature, software must be made available to editors and reviewers. We strongly encourage code deposition in a community repository (e.g. GitHub). See the Nature Portfolio guidelines for submitting code & software for further information.

Data

Policy information about availability of data

All manuscripts must include a data availability statement. This statement should provide the following information, where applicable:

- Accession codes, unique identifiers, or web links for publicly available datasets
- A description of any restrictions on data availability
- For clinical datasets or third party data, please ensure that the statement adheres to our policy

"All data necessary for recreating the figures are available at \url{https://gitlab.com/lotard/medraka_paper}. Any additional data are available on request."

Field-specific reporting

Please select the one below that is the best fit for your research. If you are not sure, read the appropriate sections before making your selection.

- [x] Life sciences
- [ ] Behavioural & social sciences
- [ ] Ecological, evolutionary & environmental sciences

For a reference copy of the document with all sections, see nature.com/documents/nr-reporting-summary-flat.pdf

Life sciences study design

All studies must disclose on these points even when the disclosure is negative.

Sample size	No sample-size calculation was performed. Every experiment was performed with N ranging from 2 to 6, so as to ensure at least duplicate reproducibility. Most of the bona fide knock-out clones were derived independently two or more times, often using different gRNAs.
Data exclusions	We excluded samples with low numbers of sequencing reads (which sample indel profiles stochastically), as well as individual indels with large deletions size (which approach read size and are therefore enriched for primer dimers).
Replication	No independent replication of results was attempted, beyond simple repeating of experiments as described in the 'Sample size' section (to wit, "every experiment was performed with N ranging from 2 to 6"). Every attempt was successful.
Randomization	We randomized position of individual clones across the experimental plate, in particular spreading out control clones.
Blinding	No blinding was performed - the novel and exploratory nature of our experimental design required data-guided analysis.

Reporting for specific materials, systems and methods

We require information from authors about some types of materials, experimental systems and methods used in many studies. Here, indicate whether each material, system or method listed is relevant to your study. If you are not sure if a list item applies to your research, read the appropriate section before selecting a response.

Materials & experimental systems

n/a	Involved in the study
[ ]	[x] Antibodies
[ ]	[x] Eukaryotic cell lines
[x]	[ ] Palaeontology and archaeology
[x]	[ ] Animals and other organisms
[x]	[ ] Human research participants
[x]	[ ] Clinical data
[x]	[ ] Dual use research of concern

Methods

n/a	Involved in the study
[x]	[ ] ChIP-seq
[ ]	[x] Flow cytometry
[x]	[ ] MRI-based neuroimaging

Antibodies

Antibodies used	"Western blots were performed using following antibodies: rabbit Parp1 (ab191217, abcam, dilution 1:8000), rabbit Lig1 (18051-1-AP, Proteintech, 1:500), rabbit Nbs1 (A301–284A, Bethyl, 1:2000), rabbit Xlf (A300-730A, Bethyl, 1:2000), mouse Actin (SC-47778, Santa Cruz, 1:200), HRP goat anti-rabbit antibody (ab205718, abcam, 1:2000) and HRP goat anti-mouse (ab205719, abcam, 1:4000) following manufacturers' recommendations."
Validation	Validation information can be found on manufacturers' websites: ab191217 - WB: mouse heart lysate; 18051-1-AP: WB species and cited specificities include mouse (but no validations provided); A301–284A - WB species reactivity: mouse, NIH 3T3 cells (also see Tsai2020); A300-730A - only human cited by manufacturer, but see Balmus2019, Bhargava2018,Tsai2020,Hung2018; SC-47778 - WB: mouse, NIH-3T3

Eukaryotic cell lines

Policy information about cell lines

Cell line source(s)	Parent cell line CB9 is described in Strogantsev 2015 Genome Biology. It was obtained from the corresponding author of the paper ie Anne Ferguson-Smith.
Authentication	We used M-FISH when establishing subclone CBA9 to check chromosomal integrity. Cell morphology and colony-forming efficiency strongly implies it remained a mouse pluripotent stem cell.
Mycoplasma contamination	The cells involved in this study tested negative for mycoplasma.

Commonly misidentified lines (See ICLAC register)	We did not use any commonly misidentified cell lines.

Flow Cytometry

Plots

Confirm that:

☒ The axis labels state the marker and fluorochrome used (e.g. CD4-FITC).

☒ The axis scales are clearly visible. Include numbers along axes only for bottom left plot of group (a 'group' is an analysis of identical markers).

☒ All plots are contour plots with outliers or pseudocolor plots.

☒ A numerical value for number of cells or percentage (with statistics) is provided.

Methodology

Sample preparation	For flow cytometric analysis, around 300,000 cells (1/6 of a near-confluent well) were collected by trypsinization, transfered to a U-bottomed 96W plate, washed once and stained for 15-60' in 50 µl buffer with relevant antibodies. After staining, cells were washed three times and analysed using a Cytoflex flow cytometer. All procedures were performed at room temperature. PBS+0.1% BSA buffer was used throughout. All centrifugations were performed for 1min at 500G.
Instrument	Cytoflex
Software	Cytoflex software ca 2017 for collection. Analysis was performed with R, using scripts deposited with this m/s.
Cell population abundance	We did not perform FACS.
Gating strategy	The simple gating strategy that was employed in this manuscript is visualized in a Supplementary Figure.

☒ Tick this box to confirm that a figure exemplifying the gating strategy is provided in the Supplementary Information.
