# Supplementary material for: Cas9-induced large deletions and small indels are controlled in a convergent fashion
Source: Nat Commun. 2022 Jun 14;13:3422. doi: 10.1038/s41467-022-30480-8 (PMC9197861; doi:10.1038/s41467-022-30480-8)
Supplement: Supplementary file 3 — Description of Additional Supplementary Files [file 41467_2022_30480_MOESM3_ESM.pdf]

Title: Supplementary Data 1

Description:

Guides
gRNAs used in the study.

Coordinates are given with respect to mm10 genome assembly.

guide mismatch in CAST genome - were there CAST-strain specific SNPs in the gRNA binding site?

region ID - unique ID of the PCR-amplified locus.

SNPs in region - were there CAST-strain specific SNPsin the amplified locus (outside of the primer binding sites)?

Clones
Detailed description of clones included in the library.

SNPs in region - were there CAST-strain specific SNPs in the amplified locus (outside of the primer binding sites)?

allele strain - origin of the allele: C=CAST, B=BL6 or U=unknown (due to lack of distinguishing SNPs or indels).

allele indel – form of the indel, D=deletion, I=insertion, X=mismatch (SAM-CIGAR format).

For clone class and definition of allele deleterious, see Methods.

mean expression negative is the average proportion of cells negative for gene expression on day 14 in the flow cytometric assay.

-log(p-value) indel profile - negative log10 FDR-corrected p-value of clones with significantly different indel profiles in a given replicate (5 or 6) from a chi-squared distribution (see Methods). ’X’ marks missing samples.

z-score indel profile - z-score of indel profiles of clones (compared to negative control clones) in a given replicate (5 or 6). ’NA’ marks missing samples.
